# Subwavelength Diffractive Optical Elements for Generation of Terahertz Coherent Beams with Pre-Given Polarization State

**DOI:** 10.3390/s23031579

**Published:** 2023-02-01

**Authors:** Vladimir Pavelyev, Svetlana Khonina, Sergey Degtyarev, Konstantin Tukmakov, Anton Reshetnikov, Vasily Gerasimov, Natalya Osintseva, Boris Knyazev

**Affiliations:** 1 Samara National Research University, 443086 Samara, Russia; 2 IPSI RAS—Branch of the FSRC “Crystallography and Photonics” RAS, 443001 Samara, Russia; 3 Budker Institute of Nuclear Physics SB RAS, 630090 Novosibirsk, Russia

**Keywords:** terahertz laser beam, diffractive optical element, free electron laser, polarization state

## Abstract

Coherent terahertz beams with radial polarization of the 1st, 2nd, and 3rd orders have been generated with the use of silicon subwavelength diffractive optical elements (DOEs). Silicon elements were fabricated by a technology similar to the technology used before for the fabrication of DOEs forming laser terahertz beams with pre-given mode content. The beam of the terahertz Novosibirsk Free Electron Laser was used as the illuminating beam. The experimental results are in good agreement with the results of the computer simulation.

## 1. Introduction

Structured laser beams [1,2,3,4,5,6,7] with controlled distribution of intensity, phase, and polarization are considered an effective tool in various applications, such as optical capture and manipulation of microparticles [8,9,10,11,12], telecommunication systems [13,14,15,16,17,18], laser material microstructuring [19,20,21,22,23], overcoming of the diffraction limit and super-resolution [24,25,26,27,28,29], plasmons excitation [30,31], and many others.

The means of diffractive optics are effective for structuring the amplitude-phase distribution of laser radiation beams [32,33,34]. Polarization transformations require more complex optical systems or anisotropic optical elements [35,36,37,38,39,40].

Structuring radiation from new sources in the terahertz range, including high-power ones, such as free-electron lasers (FELs) [41], requires optical elements designed with consideration of the features of such radiation, including wavelength and high-power density [42,43,44,45]. A good overview of the recently invented terahertz optical structures based on diffraction design is presented in [46]. The fabricated diffractive optical elements (DOEs) were used to focus [42,43,47,48,49] and split [45,50,51] the terahertz laser beam, as well as to control the transverse-mode composition of the beam [52,53,54]. In particular, silicon binary elements were used to transform the illuminating beam of a high-power free-electron terahertz laser into the Hermite–Gaussian, Laguerre–Gaussian, and Bessel single-mode beams [52,54]. However, such DOEs were used just to change the transverse mode composition without alteration of the polarization state of the illuminating beam. Note that some relevant applications of laser radiation require simultaneous control of the transverse-mode composition and polarization state of the beam [3,4,6,13,20,21]. There are well-known works on the polarization transformation of radiation in the terahertz range based on metal-dielectric metasurfaces [55,56,57]. However, all-dielectric metasurfaces [58,59,60] are preferred because they are chemically inert and are not subject to oxidation. 

Previously [59,60], the authors designed, fabricated, and examined a meta-axicon (axicon with a subwavelength period) for converting linearly polarized terahertz radiation into a second-order radially polarized beam. In this paper, we present new simulation and experimental results for the conversion of linearly polarized terahertz radiation into the first-, second-, and third-order radially polarized terahertz beams employing silicon metasurfaces based on binary subwavelength gratings with continuous ridges [61,62]. In what follows, for brevity, we will refer to these axicons as the first-order meta-axicon (MAx1), the second-order meta-axicon (MAx2), and the third-order meta-axicon (MAx3) by analogy with the term “metamaterial”.

## 2. Theoretical Description of Subwavelength Diffractive Gratings Design

It is well known [63] that a binary subwavelength grating can be represented as a uniaxial crystal. The fast axis of the crystal is perpendicular to the grooves of the lattice, and the slow axis is oriented along the grooves of the lattice. Thus, half-wave and quarter-wave plates can be made in the form of subwavelength gratings of a certain height. Ordinary and extraordinary refractive indices can be calculated with the following formulas:

(1) $$n_{eff}^{TE}={\left[Qn_{1}^{2}+\left(1-Q\right)n_{2}^{2}\right]}^{1/2}$$

(2) $$n_{eff}^{TM}={\left[Qn_{1}^{-2}+(1-Q)n_{2}^{-2}\right]}^{-1/2}$$

where *n*_1_ is the refractive index of the first medium, *n*_2_ is the refractive index of the second medium, $n_{eff}^{TE}$ is the ordinary refractive index corresponding to the direction parallel to the layers of the structure, $n_{eff}^{TM}$ is the extraordinary refractive index corresponding to the direction perpendicular to the layers of the structure. $Q=\frac{d_{1}}{d_{1}+d_{2}}$ is a fill factor ($d_{1}$ is the thickness of the silicon grating ridge and $d_{2}$ is the distance between adjacent grating ridges).

Thus, the thickness of the half-wave plate is:

(3) $$H=\lambda \sqrt{n_{1}{}^{2}+1}{\left[\sqrt{2}{\left(n_{1}-1\right)}^{2}\right]}^{-1}$$

here λ is a wavelength, *n*_2_ = 1, *Q* = 0.5.

According to Formula (3), *H* = 60 μm if *λ* = 141 μm, the refractive index of amorphous silicon *n*_1_ = *3.42.*

As practice shows, Formula (3) is not sufficiently accurate. Full-vector numerical calculation is required to determine the optimal height of the relief.

An important advantage of optical elements based on subwavelength gratings is the ability to change the directions of the slow and fast axes of the crystal by controlling the orientation of the grooves of the subwavelength grating. Thus, it is possible to create polarizing optical elements that convert the input linearly polarized radiation into beams with cylindrical polarization of various orders.

Let us consider the process of transmission through the subwavelength grating in Jones notation. Jones vector of the falling light has a view:

(4) $$E=\left(\begin{array}{c} E_{x}\\ E_{y} \end{array}\right)$$

Jones matrix of the half-wave element has a view:

(5) $$M_{\lambda /2}=\left(\begin{array}{cc} \cos2\varphi & \sin2\varphi \\ \sin2\varphi & -\cos2\varphi \end{array}\right)$$

where φ is an angle between the half-wave plate axis and the *x*-axis.

In the simplest case, when the second component of the Jones vector is equal to 0, the matrix (5) works like a rotation matrix that rotates incident polarization at an angle 2φ. Moreover, a subwavelength grating can have curved grooves causing different angles of rotation at different points of the grating. In that way, we can modulate a falling linear polarization and create a beam with spatially modulated polarization.

Radially polarized cylindrical vector beam with a topological order of *n* has the following form:

(6) $$E_{m}^{Rad}=\left(\begin{array}{c} \cos n\theta \\ \sin n\theta \end{array}\right)$$

here θ is a polar angle.

To create the beam (6), we can modify an incident linearly polarized beam with a subwavelength grating that has a matrix:

(7) $$M_{\lambda /2}=\left(\begin{array}{cc} \cos n\theta & \sin n\theta \\ \sin n\theta & -\cos n\theta \end{array}\right)$$

Comparing (7) and (5), we can conclude that the angle φ between the *x*-axis and fast axis of the grating has the following form:

(8) $$\varphi =\frac{n\theta }{2}$$

In general, the binary height of an element is determined by the formula:

(9) $$h\left(r,\varphi \right)=\frac{H}{2}\left(1+\mathrm{sign}\left(\cos\left(f\left(r,\varphi \right)\right)\right)\right)$$

where (r,φ) are the polar coordinates, sign() is the sign function and f(r,φ) is the phase of the grating.

The phase function of the element forming the beam (6) for *m* = 1 and 3 has the form [64]:

(10) $$f\left(r,\varphi \right)={\frac{4\pi }{\left(m-2\right)d}}_{0}r^{\frac{2-m}{2}}\cos\left(\frac{m-2}{2}\varphi \right)$$

For *m* = 2, the phase function of the element will have the form:

(11) $$f\left(r,\varphi \right)=\frac{2\pi r}{d_{0}}\cos\left(\frac{\varphi_{0}}{2}\right)\left(\varphi \tan\left(\frac{\varphi_{0}}{2}\right)+\ln r\right)$$

In (10) and (11) $d_{0}$ is a constant that determines the period of the grating, $\varphi_{0}$ which is an angle between the beam orientation direction and horizontal axis *x*.

**Figure 1:**
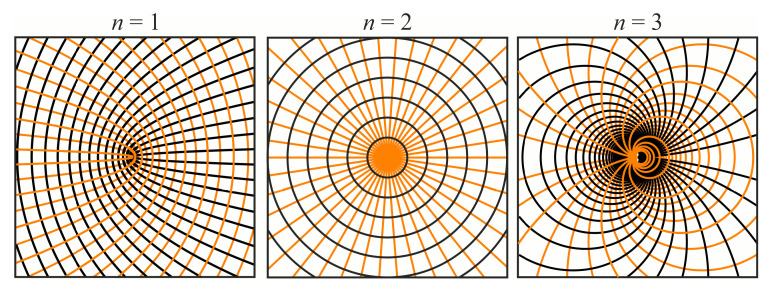
Fast (black) and slow (orange) axis of the subwavelength gratings for generation of different order (*n* = 1, 2, 3) cylindrical vector beams.

Figure 1 shows a general view of fast and slow axes for the generation of different-order radially polarized cylindrical vector beams.

Thus, we can design subwavelength gratings with curved grooves to generate cylindrical vector beams of various orders.

## 3. Design, Simulation, and Fabrication of Subwavelength Diffractive Optical Elements

The subwavelength optical element should have a height that corresponds to a half-wave plate. However, a subwavelength grating is not completely equivalent to a half-wave plate. Therefore, we use numerical simulation to find the height of the subwavelength grating that provides the best quality of the formed beam.

To find the optimal etching depth of a subwavelength grating, we consider an element of the order *n* = 2. As can be seen from Figure 1, this element has the form of an axicon and represents equidistant concentric annular ridges of a subwavelength grating.

**Figure 2:**
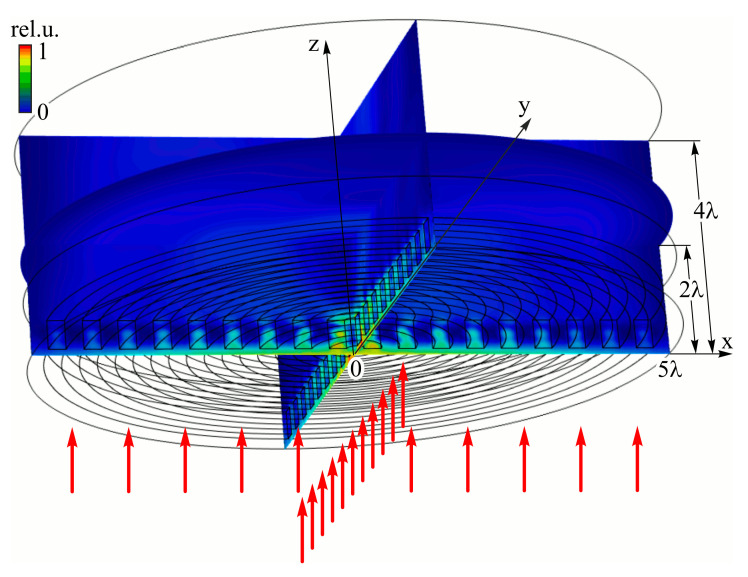
General view of numerical domain. The element is located at the bottom of the domain. Linearly polarized light incidents on the element from the bottom (red arrows). Electric field amplitude in 3D distribution of formed beam is shown in cross sections by coordinate planes (x = 0, y = 0, and z = 280 μm).

The scheme of calculation is shown in Figure 2. The calculated area has the form of a cylinder. The subwavelength grating is located at the lower part of the domain; linearly polarized light passes through the subwavelength grating from bottom to up. Electric field amplitude distributions at different cross-sections are also shown in Figure 2.

Let us consider the distribution of the electric field amplitude in the cross-section of the computational domain at different heights of the subwavelength gratings (Table 1). The first column shows the full amplitude of the electric field, the second column contains the x-component, and the third column shows the y–component.

We formulate a criterion for the quality of the beam in the following way.

Table 1 shows that horizontal and vertical polarizations form distribution patterns with four local maxima. Moreover, the distribution of the x-component has horizontally and vertically arranged pairs of maxima, and the distribution of the y-component has maxima located diagonally. Moreover, the values of the horizontally located maxima of the x-component differ from the values of the maxima located vertically. And the values of all four maxima of the distribution of the y-components are equal to each other.

**Figure 3:**
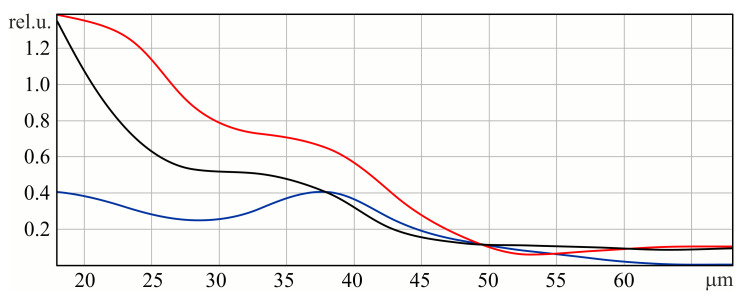
Dependence of the values of the maxima of the x-component of the horizontal (red line) and vertical (black line) pairs, as well as the values of the maximum of the y-component (blue line) on the elevation of the subwavelength grating.

Figure 3 shows the dependence of maxima values of the x-component of the horizontal and vertical pairs, as well as the values of the maximum of the y-component on the height of the relief of the subwavelength grating.

The amplitudes shown in Figure 3 have absolute values and are not normalized. According to the dynamics of these graphs, it can be seen that initially, most of the energy is contained in the X-component. This is expected because the original field is X-polarized. Increasing the height of the relief to 37 microns allows us to slightly increase the energy in the Y-component by reducing it in the X-component. However, this leaves an asymmetry in the structure of the X-components (see the first row of Table 1). Therefore, we considered a further increase in the relief height to 50 microns, where, firstly, the intersection of three graphs is observed, and, secondly, a symmetrical structure in both transverse components (see the second row of Table 1). We believe that this situation corresponds to the formation of a second-order radial polarization. Unfortunately, in this case, a significant part of the energy is lost, which is scattered on a diffraction structure with high relief.

Figure 3 shows that the three lines intersect at a relief height of 50 microns. We will choose this height for the following manufacturing of the element.

**Figure 4:**
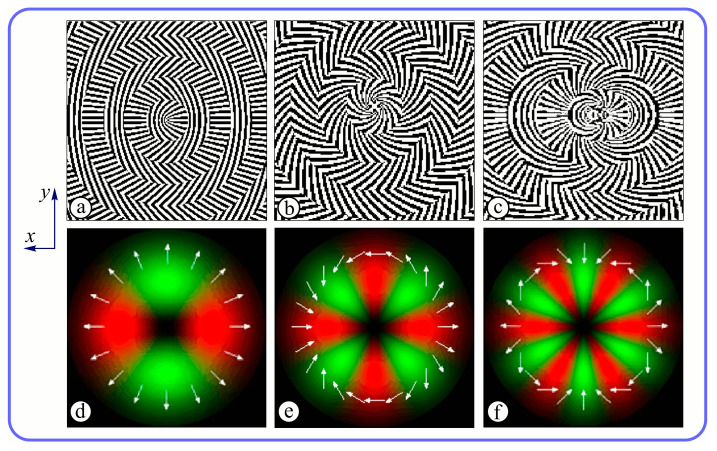
Central zones of subwavelength elements (**a**–**c**) and expected distributions of local polarization (**d**–**f**). Radiation polarized along *x*-axis is incident on elements normally to the plane of the figure.

The subwavelength elements have been designed by methods based on the rigorous light theory [64]. The following DOE parameters were chosen: the aperture diameter D = 50 mm, discretization step s = 10 μm, and wavelength λ = 141 μm. Figure 4a–c shows the calculated binary subwavelength microrelief of meta-axicons for generating terahertz beams with radial polarization of the first, second, and third orders, respectively. Also, the meta-axicons add a focusing phase to the beam (NA = 0.3). In neighboring ring-shaped Fresnel zones, subwavelength grating ridges are perpendicular to each other that provide a focusing phase in the output beam. Figure 4d–f presents the pre-given transverse distribution of the beams (red color for horizontal polarization and green color for vertical polarization).

Table 2 presents the results of the computer simulation of field distributions immediately behind the meta-axicons MAx1 (Figure 4a), MAx2 (Figure 4b), and MAx3 (Figure 4c) with added polarization analyzer rotated to the appropriate angle. The size of the domain was 1.5 mm. 

The model under consideration has the following form. Linearly polarized light falls on the element, as shown in Figure 2. The meta-axicon forms a radially polarized beam of order n with a focusing phase NA = 0.3. Next, the beam passes through an analyzing polarizer. The orientation angle of the polarizer axis relative to the horizontal axis is given in the left column. After passing the polarization analyzer, maxima are allocated for 2n sectors in accordance with the order of polarization n. Table 2 shows the rotation of the sector structure in accordance with the rotation angle of the polarization analyzer. Field distribution has a multi-ring structure because of the axicon-type focusing structure used in the metasurface.

**Figure 5:**
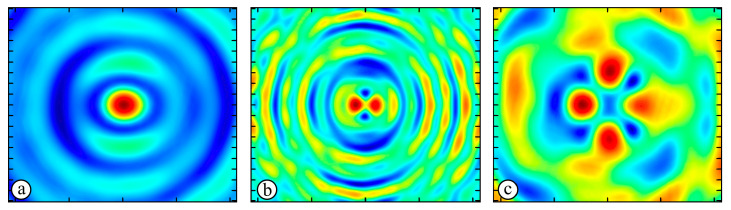
Calculated amplitude distributions at a distance of 5 wavelengths from the plane of the subwavelength elements with different polarization orders: for MAx1 (**a**), MAx2 (**b**), and MAx3 (**c**).

Figure 5 shows the results of simulating the focusing of the formed beam in the focal plane of the meta-axicons. The amplitude distributions have a special structure consisting of 2n spots, which is consistent with the results of the article [62]. In scalar theory, a ring should be in focus, but in the article [62], it was shown that the presence of a longitudinal field component significantly affects the focal distribution, which distorts the picture of full intensity.

**Figure 6:**
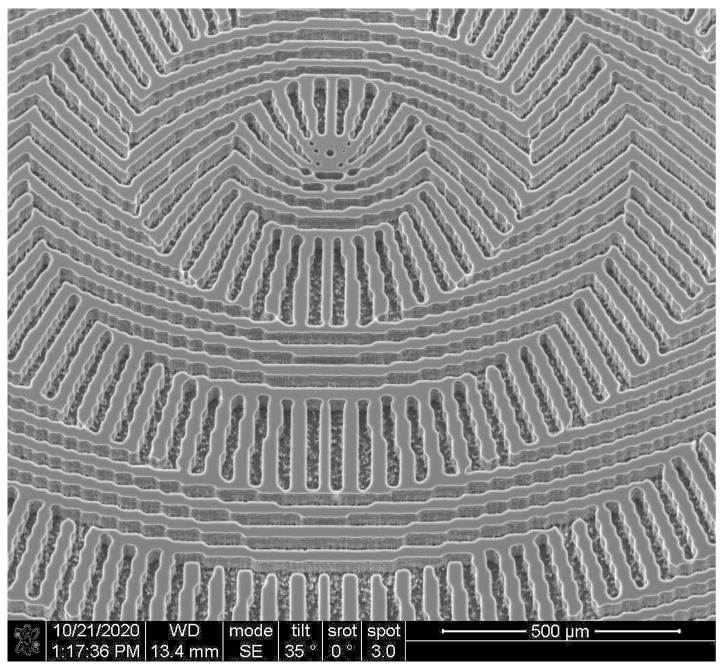
The central part of realized microrelief for MAx1 (Figure 4a) recorded by scanning electron microscopy.

The designed subwavelength elements (Figure 4a–c) were realized using the lithography technology used in [59,60] for the fabrication of terahertz subwavelength axicon. Previously, a similar technology based on the Bosch process [65] was used for the fabrication of diffractive optical elements to form laser beams with a pre-given orbital angular moment [54]. An SEM image of a realized element microrelief (Figure 4a) is shown in Figure 6.

Note that the simulation results (Table 2 and Figure 5) showed an incomplete polarization transformation, as well as the complexity of the analysis from patterns in the near diffraction zone. Therefore, to filter parasitic components, an optical scheme with a Fourier analyzer was used in the experimental study.

The following causes of incomplete polarization transformation using the meta-axicon can be distinguished. Firstly, these are Fresnel reflections due to the high refractive index (*n*_1_ = 3.42) of the meta-axicon material. Secondly, there is a non-uniform reflection due to the dependence of the refractive index on the orientation of the grating grooves. Thirdly, the thickness of the subwavelength grating also significantly affects the transmission since the grating works as a thin film. Fourth, when designing the element, an ideal averaging of the refractive indices of the lattice material and air was assumed, but the experiment was carried out in a more rigorous model, which showed a lower efficiency of the element than the ideal model.

## 4. Experimental Investigation of Subwavelength Diffractive Optical Elements

**Figure 7:**
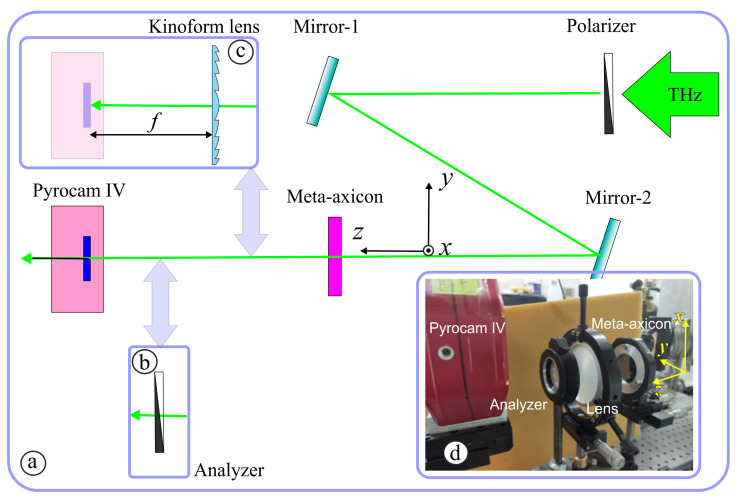
Experimental schematic. (**a**) Optical system for investigation of local polarization state of beams formed by meta-axicons. (**b**) Analyzer. (**c**) Optical Fourier transform system for study of spatial spectrum of these beams; focal length of kinoform lens is 75 mm. (**d**) Photography of the experimental setup.

The fabricated subwavelength meta-axicons (an image of one of the axicons taken with an electron microscope is shown in Figure 6) were investigated employing the terahertz radiation of the Novosibirsk free electron laser at Budker Institute of Nuclear Physics of the Siberian Branch of the Russian Academy of Sciences [41]. Laser radiation emerging as a continuous stream of 100-ps pulses with a repetition rate of 5.6 MHz was tuned to a wavelength of 141 μm. The spectral width of the radiation was about 1 μm. The Gaussian beam, *x*-polarized with a wire polarizer, incident on a meta-axicon, as shown in Figure 7. Cross-section of the beam passed through the meta-axicon was recorded (Figure 7a) by a pyroelectric camera Pyrocam IV with a matrix of 320 × 320 pixels (the size of one element was 80 μm). The total image size was 25.6 × 25.6 mm^2^. Since the camera is not sensitive to radiation polarization, in this configuration, we observed the distribution of the total beam intensity, regardless of its local polarization. For the exploration of the local polarization of the beam, an analyzer (Figure 7b) was introduced into the system. Rotating the analyzer, we were able to study the distribution of local polarization in the beam. 

In both configurations (with and without an analyzer), a polypropylene kinoform lens 80 mm in diameter with a focal length of 75 mm could be added to the optical system, as shown in Figure 7c. In this case, the recording part of the optical system turns into a Fourier analyzer, which makes it possible to study the beam spectrum behind the meta-axicon in the space of transverse wave numbers. The large diameter of the lens makes it possible to register both positive and negative orders of laser beam diffraction by the meta-axicon. A photograph of the experimental setup is shown in Figure 7d. In the case of a diffractive axicon, the expected intensity distribution in the focal plane is a ring [66].

The experiments were carried out using the axicons of the first and third orders. The purpose of the experiments was to compare the experimentally measured distributions of the local polarization of the beam after passing through the axicon illuminated by linearly polarized radiation and the polarization distribution obtained by numerical simulations. First experiments were carried out using the optical scheme of Figure 7b. They showed that behind both studied axicons, the Gaussian beam transforms into beams with a singularity near the optical axis, as predicted by the calculations (see Figure 4). The introduction of the analyzer into the optical system (Figure 7c) made it possible to see that for axicons of the first and third order, the local direction of polarization corresponds to the theoretically expected one. 

**Figure 8:**
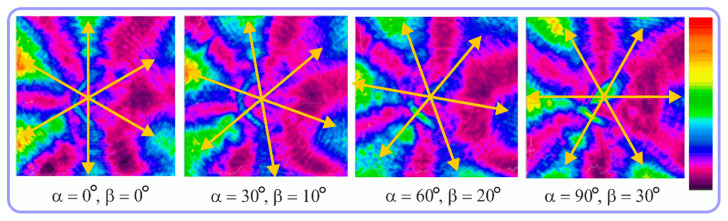
Experimentally observed intensity distribution in the beam that passed through diffractive element MAx3 and the analyzer with polarization direction α; β—observed image rotation angle shown with yellow arrows (compare with Figure 9).

**Figure 9:**
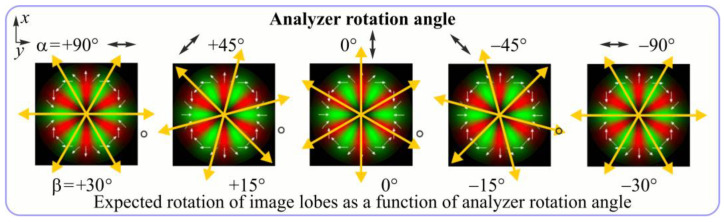
Schematics of rotation of lobes of the intensity distribution in the beam formed by third-order element MAx3 illuminated by x-polarized Gaussian beam after passing through analyzer oriented at angle α; β—angle of rotation of image.

For the MAx1 axicon at the beam periphery, as expected according to Figure 4a, the observed intensity distribution depended on the azimuthal angle α as $\cos^{2}\alpha$. In the case of the MAx3 axicon, the intensity depended on the angle as $\cos^{2}(3\alpha )$. A selection of frames recorded with Pyrocam IV for this axicon at several positions of the analyzer is shown in Figure 8. The scheme of rotation of the intensity maxima for this case is easy to understand using Figure 9. and Table 3. When the analyzer is rotated by α, the observed six annual sectors are rotated by β=α/3 (see Table 3).

**Figure 10:**
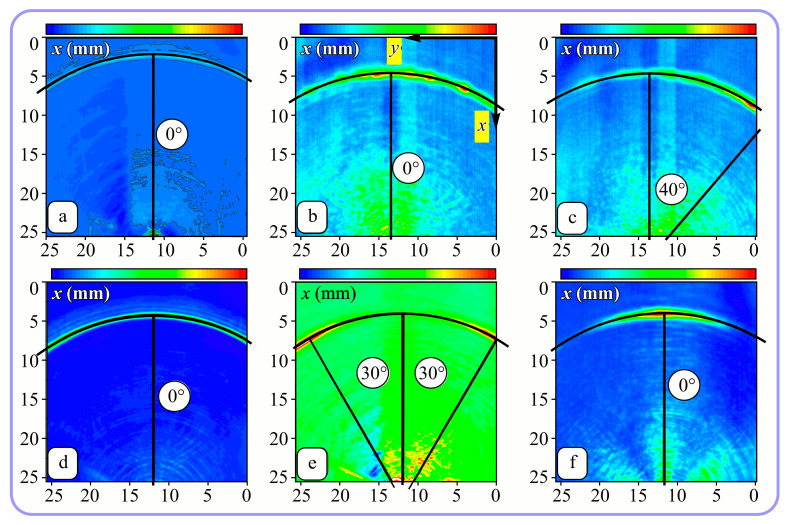
Images recorded in focal plane of lens with focal distance *f* = 75 mm; upper row: for MAx1, lower row: for MAx3. (**a**,**d**) No analyzer in the optical system. (**b**) Analyzer is oriented along *x*-axes, (**c**) analyzer has been rotated by $40^{\circ }.$ (**e**) Analyzer is oriented along *y*-axes, (**f**) analyzer has been rotated by $90^{\circ }$.

Installing a lens in the optical scheme allowed us to obtain more detailed information about the formed beams. For both axicons, in the absence of an analyzer, a uniform ring is observed in the focal plane of the lens (Figure 10a,d). This means that the beams formed are, in essence, a kind of Bessel beams of first and third orders, and they are a superposition of conically converging plane waves produced by the axicons. Since most of the beam energy is concentrated in the ring, we may assume that the diffraction efficiency is rather high. Quantitative measurements of diffraction efficiency will be carried out later. 

If an analyzer is installed between the element and the chamber, then one can observe changes in the intensity distribution associated with the polarization properties of the beam. In particular, when installing the analyzer, the intensity of the ring in the focal plane will be modulated in azimuth, and 2*n* segments will be observed, where *n* is the order of the axicon. In the case of the MAx1 axicon, two half-rings are observed (Figure 10b). When the analyzer is rotated by α = 40°, the pattern rotates by the same angle (Figure 10c). 

**Figure 11:**
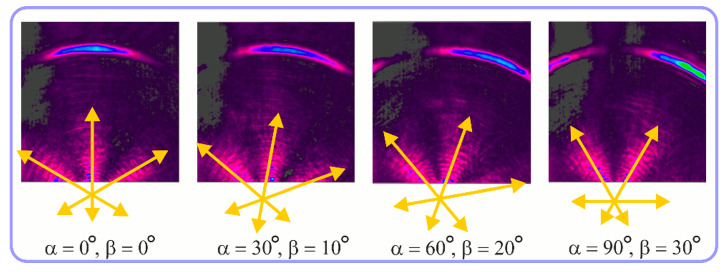
Intensity distribution of beam formed by 3rd order axicon in focal plane of lens vs. analyzer rotation.

For the MAx3 axicon, when the analyzer is rotated by an angle α, the observed six annual sectors are rotated by an angle β. For the analyzer set in the *y*-direction, we observed the segments in $\pm 30^{\circ }$ comparing to the vertical (Figure 10e). This case corresponds to Figure 9 ($\left|\alpha \right|=90^{\circ }$). Rotation of the analyzer by $90^{\circ }$ leads to the pattern shown in Figure 10f, which is schematically illustrated in Figure 9 ($\alpha =0^{\circ }$). In support of the above, at the end of the section, we present one more set of images of the beam cross-section in the focal plane of the lens, obtained by rotating the analyzer for the MAx3 axicon (Figure 11).

## 5. Conclusions

Silicon subwavelength diffractive optical elements for the generation of terahertz coherent beams with radial polarization of the first and third orders have been designed and fabricated by reactive ion etching. Methods of rigorous light theory have been used for the design of subwavelength microrelief. The fabricated elements were investigated by methods of computer simulation and natural experiments. The beam of the terahertz Novosibirsk Free Electron Laser was used as the illuminating beam (wavelength λ = 141 μm). The experimental results are in good agreement with the results of the computer simulation. It was experimentally and numerically shown that the used approach allows the generation of terahertz coherent beams with the possibility to control transverse mode content and polarization state simultaneously. This possibility is crucial for such applications as lidars [3,4], terahertz telecommunication systems with MDM [3], remote control systems for fundamental research, and so forth. In [67], it was discussed that a relatively high ratio between terahertz range wavelength and optical material structuring resolution opens an opportunity to create optical elements forming terahertz coherent fields with pre-given intensity, phase, mode content as well as polarization state. 

## Figures and Tables

**Table 1:** Electric field distributions in the cross-section of the computational domain at different heights of the subwavelength gratings.

Height of the Relief (μm)	E-Field Components		
	Full Amplitude	x-Component	y-Component
40	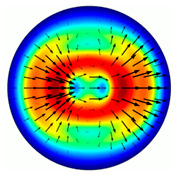	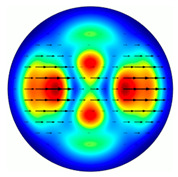	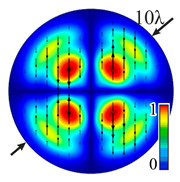
50	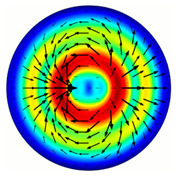	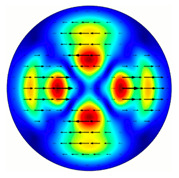	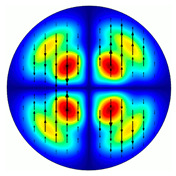
60	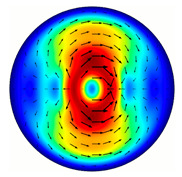	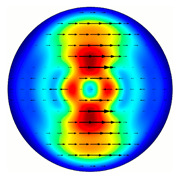	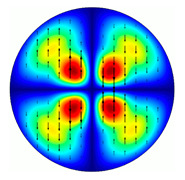

**Table 2:** Results of computer simulation of field distributions immediately behind the meta-axicons that form radially polarized beams with the order of 1, 2, and 3. Beams pass through an analyzer rotating clockwise; the incident beam is polarized along the *x*-axis.

Analyzer Rotation Angle (Deg)	Radial Polarization Order		
	*n* = 1	*n* = 2	*n* = 3
0	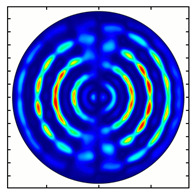	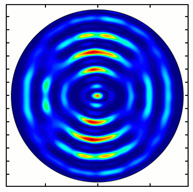	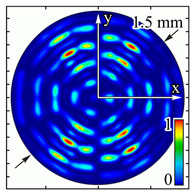
30	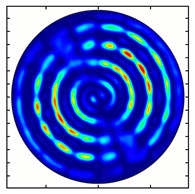	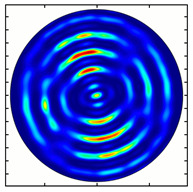	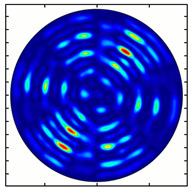
60	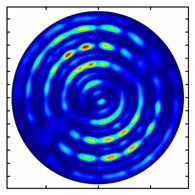	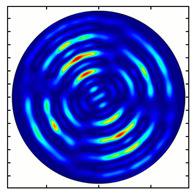	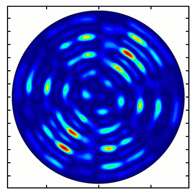
90	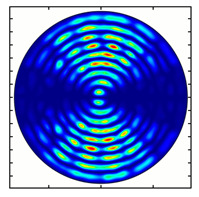	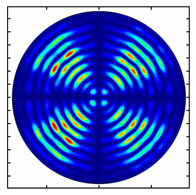	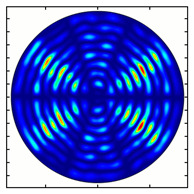
120	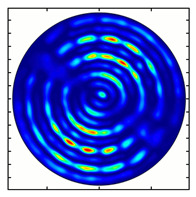	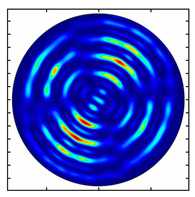	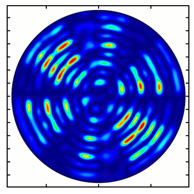
150	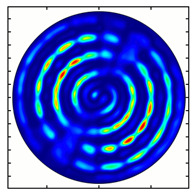	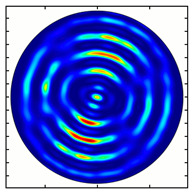	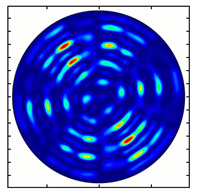
180	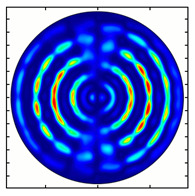	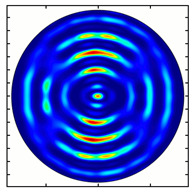	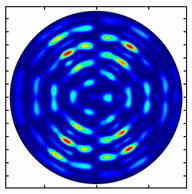

**Table 3:** Rotation of sectors of intensity lobes as a function of analyzer rotation angle for axicon of third order.

α	0	10	20	30	40	50	60	70	80	90
β=α/3	0	3	7	10	13	17	20	23	27	30

## Data Availability

The data that support the findings of this study are available from the authors upon reasonable request.
